# A protocol for a systematic review on the impact of unpublished studies and studies published in the gray literature in meta-analyses

**DOI:** 10.1186/2046-4053-2-24

**Published:** 2013-05-02

**Authors:** Christine Schmucker, Annette Bluemle, Matthias Briel, Susan Portalupi, Britta Lang, Edith Motschall, Guido Schwarzer, Dirk Bassler, Katharina F Mueller, Erik von Elm, Joerg J Meerpohl

**Affiliations:** 1 German Cochrane Center, Institute of Medical Biometry and Medical Informatics, University Medical Center Freiburg, 79110 Freiburg, Germany; 2 Basel Institute for Clinical Epidemiology and Biostatistics, University Hospital Basel, Basel, 4031, Switzerland; 3 Department of Clinical Epidemiology and Biostatistics, McMaster University, Hamilton, Canada; 4 Institute of Medical Biometry and Medical Informatics, University Medical Centre Freiburg, Freiburg, 79104, Germany; 5 Centre for Paediatric Clinical Studies, University Medical Center Tuebingen, Tuebingen, 72070, Germany; 6 Cochrane Switzerland, IUMSP, University Hospital Lausanne, Lausanne, 1005, Switzerland

**Keywords:** Publication bias, Gray literature, Unpublished studies, Meta-analyses, The OPEN project

## Abstract

**Background:**

Meta-analyses are particularly vulnerable to the effects of publication bias. Despite methodologists’ best efforts to locate all evidence for a given topic the most comprehensive searches are likely to miss unpublished studies and studies that are published in the gray literature only. If the results of the missing studies differ systematically from the published ones, a meta-analysis will be biased with an inaccurate assessment of the intervention’s effects.

As part of the OPEN project (http://www.open-project.eu) we will conduct a systematic review with the following objectives:

▪ To assess the impact of studies that are not published or published in the gray literature on pooled effect estimates in meta-analyses (quantitative measure).

▪ To assess whether the inclusion of unpublished studies or studies published in the gray literature leads to different conclusions in meta-analyses (qualitative measure).

**Methods/Design:**

*Inclusion criteria:* Methodological research projects of a cohort of meta-analyses which compare the effect of the inclusion or exclusion of unpublished studies or studies published in the gray literature.

*Literature search:* To identify relevant research projects we will conduct electronic searches in *Medline*, *Embase* and *The Cochrane Library*; check reference lists; and contact experts.

*Outcomes:* 1) The extent to which the effect estimate in a meta-analyses changes with the inclusion or exclusion of studies that were not published or published in the gray literature; and 2) the extent to which the inclusion of unpublished studies impacts the meta-analyses’ conclusions.

*Data collection:* Information will be collected on the area of health care; the number of meta-analyses included in the methodological research project; the number of studies included in the meta-analyses; the number of study participants; the number and type of unpublished studies; studies published in the gray literature and published studies; the sources used to retrieve studies that are unpublished, published in the gray literature, or commercially published; and the validity of the methodological research project.

*Data synthesis:* Data synthesis will involve descriptive and statistical summaries of the findings of the included methodological research projects.

**Discussion:**

Results are expected to be publicly available in the middle of 2013.

## Background

A meta-analyses as part of a systematic review aims to provide a thorough, comprehensive and unbiased account of the literature [1,2]. However, potentially important studies could be missing from a meta-analysis because of selective publication and inadequate dissemination of results. Despite methodologists’ best efforts to locate all eligible evidence for a given topic the most comprehensive searches are likely to miss unpublished studies and studies that are not commercially published and, therefore, are not indexed in respective databases (so called gray literature, such as conference abstract, dissertations, policy documents, book chapters). If the results from missing studies differ systematically from the published data, a meta-analysis may become biased with an inaccurate assessment of the intervention’s effects. For instance, positive, significant findings are more likely to be published than non-significant findings, and a meta-analysis which is based mainly on published literature may end up overestimating the efficacy of the intervention [3-5].

However, the impact of gray literature and unpublished studies on the conclusions of meta-analyses has not been comprehensively clarified. For example, there is some evidence that suggests that published randomized controlled trials (RCTs) tend to be larger and show an overall greater treatment effect than gray trials [6]. But the identification of relevant unpublished studies or studies published in the gray literature and their inclusion in meta-analyses can be particularly time-consuming and challenging. There is also some controversy as to whether unpublished studies and studies published in the gray literature should be included in meta-analyses because they might be incomplete and their methodological quality (validity) can be difficult to assess. A publication by Cook and colleagues in 1993 showed that 78% of authors of meta-analyses felt that unpublished studies should be included in meta-analyses compared to only 47% of journal editors [7]. Therefore, research is needed to help assess the potential implications for reviewers of not including gray literature and unpublished studies in meta-analyses of health care interventions.

### Objectives

In terms of the above mentioned controversies regarding the inclusion of unpublished studies and studies published in the gray literature on the results of meta-analyses, we will conduct a systematic review with the following objectives:

▪ To assess the impact of studies that were not published or only published in the gray literature on pooled effect estimates in meta-analyses (quantitative measure)

▪ To assess whether the inclusion of unpublished studies or studies published in the gray literature impacts the conclusions of meta-analyses (qualitative measure)

This systematic review will be part of the OPEN Project (To **O**vercome failure to **P**ublish n**E**gative fi**N**dings) which was developed with the goal of elucidating the scope of non-publication of studies through a series of systematic reviews. In an earlier issue of this journal (‘Systematic Reviews’), our group has already published a protocol for a systematic review which evaluates the extent of non-publication of research studies, which were approved by ethics committees, registered in trial registries or presented as conference abstracts [8].

## Methods/Design

### Search methods for identification of methodological research projects

To identify the relevant research evidence we will conduct electronic literature searches in the following databases: *Ovid Medline* (1946 to present), *Ovid Medline* Daily Update, *Ovid Medline* in process & other non-indexed citations, *Ovid Embase* (1980 to present), *The Cochrane Library* (most current issue) and *Web of Science*. No language restrictions will be applied.

In addition, the bibliographies of any eligible articles identified will be checked for additional references and citation searches will be carried out for all included references using ISI Web of Knowledge.

A search strategy for the electronic literature search in *Ovid Medline* has already been designed with the support of a librarian/information specialist. This strategy was translated as appropriate for the other databases (for the full search strategies see Appendix A). In addition, we will contact various experts in the field for further eligible studies.

### Data collection and analyses

#### Selection of methodological research projects

A methodological research project will be considered eligible for inclusion in this systematic review if it reviews a cohort of meta-analyses (that is, more than one meta-analyses) that:

▪ compare pooled effect estimates of meta-analyses of health care interventions according to publication status (that is, published versus unpublished studies or gray literature) or

▪ examine whether the inclusion of unpublished studies or gray literature impacts the overall findings or conclusions of a meta-analyses

We will consider ‘published’ articles to be manuscripts that appeared in peer-reviewed journals. Our working definition of gray literature will correspond to the definitions used by the authors of eligible methodological research projects and which also conforms to the definition of ‘gray literature’ described earlier in this protocol (see Background). A meta-analysis is defined as the calculation of a summary estimate of treatment effect by pooling the results of two or more studies.

#### Data extraction

A specifically designed data extraction form will be developed and two reviewers will independently extract all relevant data from eligible methodological research projects. The following information will be collected:

1. Characteristics of the methodological research project

a. Baseline data (for example, author names, affiliation, language and year of publication, funding, type of report (for example, full publication, abstract))

a. Area of health care/medical specialty

a. Number of meta-analyses included

2. Characteristics of the meta-analyses included in the methodological research project

a. Type of meta-analyses (for example, individual patient data meta-analyses)

a. Number of studies included in meta-analyses (overall, median, range)

a. Number of participants included in meta-analyses (overall, median, range)

a. Main purpose of meta-analyses (efficacy versus safety)

a. Source used to retrieve unpublished studies, studies published in the gray literature and published studies

3. Characteristics of the studies included in the meta-analyses

a. Number of unpublished studies, studies published in the gray literature and published studies

a. Number of participant in unpublished studies, in studies published in the gray literature and in published studies

a. Number of statistically significant positive or negative unpublished studies, studies published in the gray literature and published studies

a. Type of unpublished studies (for example, RCTs, observational studies), studies published in the gray literature (for example, abstracts, dissertation, letter, book chapters) and published studies (for example, RCTs, observational studies)

a. Year of publication of unpublished studies, studies published in the gray literature and published studies

a. Language and country of unpublished studies, studies published in the gray literature and published studies

a. Funding source of unpublished studies, studies published in the gray literature and published studies

a. Type of data source in which gray, unpublished and published studies were identified

a. Methodological quality (for example, blinding, follow-up time, sample size calculation) of unpublished studies, studies published in the gray literature and published studies (this aspect can only be evaluated if the methodological research project provides enough information)

#### Assessment of validity

We will systematically consider the validity and generalizability of the identified evidence provided by each of the methodological research projects by evaluating the following aspects:

1. Internal validity:

a. Role of confounding factors: The results of published studies may differ from those of unpublished studies because of factors other than publication status, such as study design, type of participants, characteristics of the intervention, and methodological quality; in this context, did the researcher of the meta-analyses select comparison groups that were matched (for example, did the unpublished studies or studies published in the gray literature share similar aims, designs, and sample sizes as the published ones)?; if not, were suitable adjustments for potentially confounding factors made?

a. Definition of publication status: Are explicit criteria given to categorize or define unpublished studies, studies published in the gray literature and published studies?

a. Selection process: Are search criteria given to identify unpublished studies, studies published in the gray literature and published studies?

2. External validity (generalizability):

a. Did the researcher of the methodological research project select a broad-ranging sample of meta-analyses that reflect the current literature in the field of interest (for example, in terms of size, diversity of topic)?

a. How was the sample determined (for example, random sample)?

a. Did two researchers carry out data extraction independently?

a. Did the researchers provide a complete dataset (regarding the characteristics of the methodological research project and included meta-analyses)?

#### Outcome measures

1. The extent to which the effect estimate in a meta-analyses changes with the inclusion or exclusion of unpublished studies and gray literature *(*quantitative measurement*).* If possible, we will calculate a ratio of risks or odds ratios between the results of unpublished studies and studies published in the gray literature and the results of published studies and estimate the percentage change (pooled risk ratio from unpublished studies and gray literature divided by pooled risk ratio from published studies). A weighted pooled overall estimate will be calculated taking into account number of studies, participants and events.

2. The impact of the inclusion of unpublished studies or studies published in the gray literature on conclusions of meta-analyses (qualitative measurement). The impact will be estimated by calculating the proportion of meta-analyses which show a change in their conclusions according to publication status of the included studies; categorization will be as follows:

a. Change from negatively significant to positively significant

a. Change from inconclusive to positively significant

a. Change from positively significant to inconclusive

a. Change from negatively significant to inconclusive

a. Change from inconclusive to negatively significant

a. Change from positively significant to negatively significant

a. Change from not clinical relevant to clinical relevant

a. Change from not clinical relevant to inconclusive

a. Change from clinical relevant to inconclusive

a. Change from clinical relevant to not clinical relevant

a. Change from inconclusive to clinical relevant

a. Change from inconclusive to not clinical relevant

Significance and clinical relevance will be defined according to the definitions provided in the methodological research project.

#### Unit of analyses issues

The anticipated unit of analyses is the meta-analyses included in the methodological research project.

#### Assessment of heterogeneity

Heterogeneity for pooled outcome measures will be assessed by standard methods including Chi^2^-test and calculation of the I^2^ value [9].

#### Assessment of reporting biases

Funnel plots will be used to assess the association between point estimates of log odds ratio (a measure of extent of association between meta-analyses’ characteristics and change in summary estimates) and a measure of precision if more than ten methodological research projects provide necessary information. Funnel plots will be visually assessed and appropriate formal statistical tests following recommendations formulated by Sterne *et al*. will be used to test for asymmetry [5]. In the instance of suspected reporting bias authors will be contacted.

#### Data synthesis

Data synthesis will involve a combination of descriptive and statistical summaries of the impact of the inclusion or exclusion of unpublished studies and gray literature on the results of meta-analyses (identified by methodological research projects).

The decision on whether or not to combine the results of the included methodological research projects will depend on the assessment of heterogeneity. Where methodological research projects will be judged to be sufficiently homogenous in their design a meta-analyses of these research projects will be carried out. The estimated ratios of unpublished and published in the gray literature only versus published treatment effects generated from each methodological evaluation will then be used to summarize the overall difference in risk ratios between unpublished and published in the gray literature only and published studies. The 95% confidence interval for the combined effect will be estimated using a random effects model.

#### Subgroup analyses and investigation of heterogeneity

The following subgroup analyses are planned:

1. On the level of the methodological research project

a. Number of meta-analyses included in the methodological research project

a. Number of participants included in the methodological research project

2. On the level of the meta-analyses

a. Number of studies (unpublished studies versus studies published in the gray literature versus both)

a. Number of participants included in studies (unpublished studies versus studies published in the gray literature versus both)

a. Design of studies (unpublished studies versus studies published in the gray literature versus both)

a. Source of database: gray literature (for example, conference abstracts or research letters) published in an easily accessible database versus unpublished studies for which immense efforts are required to be identified (for example, contact with pharmaceutical industry)

a. Type of research work (drug versus non-drug studies, clinical research versus basic research)

a. Area of health care

#### Sensitivity analyses

No sensitivity analyses are planned. However, should the instance arise where a methodological research project with doubtful eligibility is identified (as determined by individual review and analyses of the validity of the methodological research project), sensitivity analyses may be undertaken. Possible sensitivity analyses may be based on the following:

▪ Methodological quality/validity of the methodological research project; only methodological research projects with low risk of bias will be considered. A methodological research project will be of high risk of bias when the external validity and/or the validity of the included meta-analyses are doubtful.

## Discussion

This systematic review seeks to comprehensively synthesize the growing body of research that is related to the impact of including unpublished studies and studies published in the gray literature in meta-analyses. By considering multiple characteristics and potential confounders related to unpublished studies and studies published in the gray literature, we hope to identify sufficient evidence to conclude whether (or to what extent) inclusion of unpublished studies and studies published in the gray literature has an impact on the pooled effect estimates and the conclusions from a meta-analyses. The findings, including risk factors for unpublished studies and studies published in the gray literature, will have important implications for researchers conducting meta-analyses since they need to be informed about the impact and extent of (not) including unpublished and gray studies in meta-analyses. In addition, this systematic review in combination with the results of other systematic reviews that are part of the OPEN Project will serve to raise awareness about the impact of publication bias and the complexity of this issue. These reviews will also serve as a foundation for a recommendations workshop which will enable key members of the biomedical research community (for example, funders, research ethics committees, and journal editors) to develop future policies and guidelines to lessen the frequency of non-publication and related biases.

We acknowledge that more than half of all systematic reviews do not involve meta-analysis in their analyses. Despite the fact that our main outcomes focus on the impact of unpublished and gray studies on pooled effect estimates in meta-analyses, our findings will also be valuable for systematic reviews. It is obvious, if we find a statistical difference in the pooled effect estimates in meta-analyses, it is also likely that gray and unpublished literature impacts descriptive results of systematic reviews. Beside effect estimates, we will also evaluate differences in the methodological quality and study characteristics (such as number of participants, language or methodological quality) between unpublished, gray and published studies. These results will also be valuable for systematic reviews to appraise the potential impact of publication bias.

## Appendix A

### Search Strategy for OvidSP MEDLINE (search strategy will be adapted for other databases)

Line 10 to 14 and 16 to 35 are not shown because they are part of the search strategy for our 1^st^ systematic review [8] and, therefore, have no consequence for this search.

1. exp Publishing/sn

2. *publishing/

3. publication bias/

4. selection bias/

5. exp manuscripts as topic/

6. ((data or finding? or information or evidence or study or studies or trial? or paper? or article? or report* or literature or work or manuscript? or abstract* or result?) adj6 (unpublish* or un-publish* or unreport* or un-report* or nonpublish* or non-publish* or nonpublicat* or non-publicat* or (publication? adj3 rate?) or "not publish*")).ti,ab.

7. (underreport* or under-report* or selective report* or selective publish* or selective publicat* or (final* adj2 (report* or publish* or publicat* or manuscript? or paper? or article?)) or (full? adj2 (report* or publish* or publicat* or manuscript? or paper? or article?)) or (subsequent* adj2 (report* or article? or paper? or publi* or manuscript?)) or (sub-sequent* adj2 (report? or article? or paper? or publi* or manuscript?)) or (complete* adj2 (report* or article? or paper? or publish* or publicat* or manuscript?))).ti,ab.

8. (bias* adj3 (publish* or publicat*)).ti,ab.

9. or/1-8

15. exp animals/ not humans/

36. meta-analysis as topic/

37. Guidelines as Topic/ or Practice Guidelines as Topic/

38. exp Clinical Trials as Topic/

39 meta-analysis.pt.

40. (guideline or practice guideline).pt.

41. (guideline? or metaanaly* or meta-analy* or metanaly* or meta-synthe* or metasynthe* or meta-regressi* or metaregressi*).ti,ab.

42. (systematic* adj3 (review* or overview*)).ti,ab.

43. exp Technology Assessment, Biomedical/

44. (health technology assessment? or HTA).ti,ab.

45. or/36-44

46. 9 and 45

47. 46 not 15

48. ((implication? or impact? or influenc* or effect? or differen*) adj6 (publication bias* or unpublish* or un-publish* or unreport* or un-report* or nonpublish* or non-publish* or nonpublicat* or non-publicat* or "not publish*")).ti,ab.

49. ((implication? or impact? or influenc* or effect? or differen*) adj6 (selective report* or selective publish* or selective publicat* or (final* adj2 (report* or publish* or publicat* or manuscript? or paper? or article?)) or (full? adj2 (report* or publish* or publicat* or manuscript? or paper? or article?)) or (subsequent* adj2 (report* or article? or paper? or publi* or manuscript?)) or (sub-sequent* adj2 (report? or article? or paper? or publi* or manuscript?)) or (complete* adj2 (report* or article? or paper? or publish* or publicat* or manuscript?)))).ti,ab.

50. ((unpublish* or un-publish* or unreport* or un-report* or nonpublish* or non-publish* or nonpublicat* or non-publicat* or "not publish*") adj6 publish*).ti,ab.

51. (underreport* or under-report* or selective report* or selective publish* or selective publicat* or (final* and (report* or publish* or publicat* or manuscript? or paper? or article?)) or (full? and (report* or publish* or publicat* or manuscript? or paper? or article?)) or (subsequent* and (report* or article? or paper? or publi* or manuscript?)) or (sub-sequent* and (report? or article? or paper? or publi* or manuscript?)) or (complete* and (report* or article? or paper? or publish* or publicat* or manuscript?))).ti.

52. (unpublish* or un-publish* or unreport* or un-report* or nonpublish* or non-publish* or nonpublicat* or non-publicat* or "not publish*" or bias*).ti.

53. or/48-52

54. 47 and 53

55. 47 and (3 or 4)

56. 47 and (36 or 37)

57. 56 and (6 or 7)

58. *meta-analysis as topic/

59. *Guidelines as Topic/ or *Practice Guidelines as Topic/

60. 47 and (58 or 59)

61. (6 or 7) and (3 or 8)

62. 54 or 55 or 57 or 60 or 61

63. (unpublish* or un-publish* or unreport* or un-report* or nonpublish* or non-publish* or nonpublicat* or non-publicat* or "not publish*").ti,ab.

64. 7 or 63

65. 62 and 64

66. 36 or 37

67. 3 and 66

68. 65 or 67

69. 68 not 15

70. remove duplicates from 69

## Appendix B

### OPEN Consortium

Table 1 shows OPEN Consortium.

**Table 1:** OPEN Consortium

**Contributor**	**Participating Institution**
Antes, Gerd	German Cochrane Center, Institute of Medical Biometry and Medical Informatics University Medical Center, Freiburg, Germany
Bassler, Dirk	Center for Pediatric Clinical Studies, University Medical Center Tuebingen, Germany
Bertele, Vittorio	Department of Epidemiology, Mario Negri Institute for Pharmacological Research, Italy
Bonfill, Xavier	The Clinical Epidemiology & Public Health Department at the Hospital de la Santa Creu i Sant Pau, Spain
Bouesseau, Marie-Charlotte	World Health Organization, Geneva, Switzerland
Boutron, Isabelle	INSERM U738 research unit, Paris Descartes University, Paris, France
Gallus, Silvano	Department of Epidemiology, Mario Negri Institute for Pharmacological Research, Italy
Garattini, Silvio	Department of Epidemiology, Mario Negri Institute for Pharmacological Research, Italy
Ghassan, Karam	World Health Organization, Geneva, Switzerland
La Vecchia, Carlo	Department of Epidemiology, Mario Negri Institute for Pharmacological Research, Italy
Lang, Britta	German Cochrane Center, Institute of Medical Biometry and Medical Informatics University Medical Center, Freiburg, Germany
Littmann, Jasper	CELLS (Centre for Ethics and Law in Life Sciences), Hannover Medical Scholl, Hannover, Germany
Kleijnen, Jos	Kleijnen Systematic Reviews Ltd., York, United Kingdom
Kulig, Michael	Federal Joint Committee, Berlin, Germany
Malicki, Mario	University of Split School of Medicine, Split, Croatia
Marusic, Ana	University of Split School of Medicine, Split, Croatia
Meerpohl, Joerg	German Cochrane Center, Institute of Medical Biometry and Medical Informatics University Medical Center, Freiburg, Germany
Mueller, Katharina Felicitas	Center for Pediatric Clinical Studies, University Medical Center Tuebingen, Germany
Pardo, Hector	The Clinical Epidemiology & Public Health Department at the Hospital de la Santa Creu i Sant Pau, Spain
Perleth, Matthias	Federal Joint Committee, Berlin, Germany
Ravaud, Philippe	INSERM U738 research unit, Paris Descartes University, Paris, France
Reis, Andreas	World Health Organization, Geneva, Switzerland
Schmucker, Christine	German Cochrane Center, Institute of Medical Biometry and Medical Informatics University Medical Center, Freiburg, Germany
Schwarzer, Guido	German Cochrane Center, Institute of Medical Biometry and Medical Informatics University Medical Center, Freiburg, Germany
Strech, Daniel	CELLS (Centre for Ethics and Law in Life Sciences), Hannover Medical Scholl, Hannover, Germany
Trinquart, Ludovic	INSERM U738 research unit, Paris Descartes University, Paris, France
Urrútia, Gerard	The Clinical Epidemiology & Public Health Department at the Hospital de la Santa Creu i Sant Pau, Spain
Von Elm, Erik	German Cochrane Center, Institute of Medical Biometry and Medical Informatics University Medical Center, Freiburg, Germany
Wager, Elizabeth	Sideview, Princes Risborough, United Kingdom
Wieland, Alexandra	Federal Joint Committee, Berlin, Germany
Wolff, Robert	Kleijnen Systematic Reviews Ltd., York, United Kingdom

## Competing interests

We declare that all authors and contributing members have no competing interests.

## Authors’ contributions

JM is the lead researcher of this project. JM and CS, along with EvE and SP, developed the methodologies of the systematic review protocol and led the writing of the protocol. EM designed the search strategy. DB, MB and GS contributed significantly to the writing and revision of the protocol. All authors critically revised the protocol and read and approved the final version.
